# Supplementary material for: Identification of Freezing-Responsive microRNAs and Their Targets in Chinese Jujube by Small RNA and Degradome Sequencing
Source: Int J Mol Sci. 2026 Sep 4;27(17):7912. doi: 10.3390/ijms27177912 (PMC13566890; doi:10.3390/ijms27177912)
Supplement: Supplementary file 1 [file ijms-27-07912-s001.zip › Supplementary Material S2/novel_miR_18.pdf]

Provisional ID : novel_miR_18
Score total : 0.4
Score for star read(s) : -1.3
Score for read counts : 0
Score for mfe : 0.1
Score for randfold : 1.6
Score for cons. seed :
Total read count : 337
Mature read count : 337
Loop read count : 0
Star read count : 0

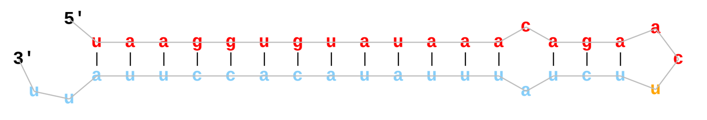

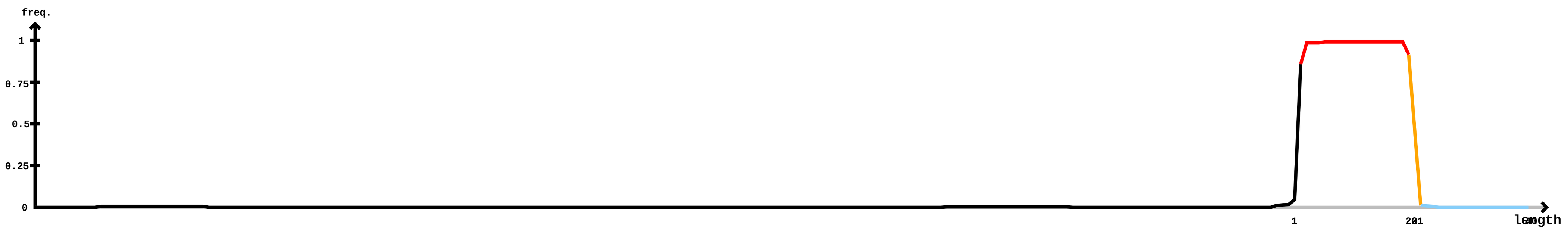

Mature Star

5'- guggaaaauauaccggauuaauugauuagaauuggaauuaauuguuaagucaucuauacauuuuucuacucaugcuuuuuuauugauuuuguaucuuuuucauccuacucuuguuaucuauuuaauuuugaauuaaauauuuuuuuguuucauuagaacuugauuauauuugguucuuauucaauuucaaucagcagaagcaaacaaaauaagguguauaaacagaacuucuauuuauacaccuuauuuu -3' exp

((((((((((((...(((((((((((((........))))))))))....)))...))))...))))))))...(((((((((((((..(((....................((((......))))...((((((((((..((..((((.....))))..))..))))))))))).....)))..))))).)).))))))).... (((((((((((((((.(((...))).))))))))))))))).

read	reads	mm	sample
.........auaccggauuaauugauu...	1	0	JCK-2
.........auaccggauuaauugauu...	1	0	LT-3
...cauuagaacuugaAuauauuu...	1	1	LCK-3
...aaaauaagguguauaaacagaa...	1	0	JCK-1
...aaaauaagguguauaaacagaa...	2	0	JCK-3
...aaaauaagguguauaaacagaac...	1	0	JCK-3
...aaauaagguguauaaacagaac...	1	0	LT-1
...aauaagguguauaaacagaa...	1	0	LT-3
...auaagguguauaaacagaac...	4	0	LT-2
...auaagguguauaaacagaac...	2	0	LT-3
...auaagguguauaaacagaac...	1	0	JCK-3
...auaagguguauaaacagaac...	2	0	LT-1
...auaagguguauaaacagaacuu...	1	0	JCK-2
...uaagguguauaaacagaC...	5	1	LCK-3
...uaagguguauaaacagaa...	7	0	LT-3
...uaagguguauaaacagaa...	3	0	JT-2
...uNagguguauaaacagaa...	1	1	JT-2
...uaagguguauaaacagaa...	2	0	LT-2
...uaagguguauaaacagaa...	1	0	JCK-1
...uaagguguauaaacagaa...	1	0	JT-3
...uaagguguauaaacagaa...	2	0	JCK-2
...uaagguguauaaacagaac...	26	0	LT-2
...uaagguguauaaacagaac...	62	0	LT-3
...uaagguguauaaacagaac...	49	0	LCK-3
...Caagguguauaaacagaac...	1	1	JCK-1
...uaagguguauaaacagaac...	14	0	JT-1
...uaagguguauaaacagaac...	17	0	JCK-2
...uaagguguauaaacagaac...	42	0	LT-1
...uaagguguauaaacagaac...	17	0	JCK-1
...uaagguguauaaacagaac...	26	0	JCK-3
...uCagguguauaaacagaac...	1	1	LT-1
...uaagguguauaaacagaacu...	1	0	LT-1
...uaagguguauaaacagaacA...	2	1	LT-1
...aagguguauaaacagaac...	10	0	LCK-3

Mature Star

guggaaaauauaccggauuaauugauuagaauuggaauuaauuguuaagucaucuauacauuuuucuacucaugcuuuuuuauugauuuuguaucuuuuucauccuacucuuguuaucuauuuaauuuugaauuaaauauuuuuuuguuucauuagaacuugauuauauuugguucuuauucaauuucaaucagcagaagcaaacaaaauaagguguauaaacagaacuucuauuuauacaccuuauuuu

....aagguguauaaacagaac....	8	0	LT-1
....aagguguauaaacagaac....	6	0	LT-3
....aagguguauaaacagaac....	9	0	LT-2
....aagguguauaaacagaac....	2	0	JCK-3
....aagguguauaaacagaacu....	2	0	LT-1
....aagguguauaaacagaacA....	1	1	JCK-1
....aagguguauaaacagaacA....	2	1	JCK-2
....aagguguauaaacagaacA....	1	1	JCK-3
....aagguguauaaacagaacu....	2	0	LT-3
....aagguguauaaacagaCcuuc....	1	1	JCK-2
....guguauaaacagaacuucu....	2	0	LT-1
